# Highlight: Adaptations That Rule the Night

**DOI:** 10.1093/gbe/evaa199

**Published:** 2020-10-16

**Authors:** Casey McGrath

As the only birds with a nocturnal, predatory lifestyle, owls occupy a unique niche in the avian realm. Hunting prey in the dark comes with a number of challenges. Owls have evolved several features that leave them well suited to this task, combining raptorial traits like acute vision and sharp talons with nocturnal adaptations such as enhanced hearing and night vision. In a recent article in *Genome Biology and Evolution* titled “Genomic evidence for sensorial adaptations to a nocturnal predatory lifestyle in owls,” Pamela Espíndola-Hernández, a doctoral student at the Max Planck Institute for Ornithology, and colleagues report the results of a genome-wide scan to uncover the genetic and selective mechanisms that underlie the owls’ particular adaptations (Espíndola-Hernández et al. 2020). In addition to confirming the important role of the visual and auditory systems, the study, which was overseen by Dr Bart Kempenaers and Dr Jakob Mueller, in collaboration with Dr Martina Carrete at the Universidad Pablo de Olavide in Spain, suggests the existence of an unusual adaptation not yet described in birds, shedding new light on the evolutionary history of this nighttime predator. Specifically, the authors propose that selection has acted on epigenetic mechanisms to package the DNA in retinal cells in such a way that it acts as a light-channeling lens to enhance photoreception.

The majority of birds have a diurnal lifestyle, meaning they are primarily active during the day. Owls, comprising the avian order Strigiformes, are thought to have diverged from their sister group, the Coraciimorphs (including mousebirds, woodpeckers, and kingfishers), during the Paleocene, when a radiation of small mammals may have led to greater availability of nocturnal prey. To better take advantage of this nightly feast, owls presumably retained predatory features shared with other raptors like eagles and hawks. At the same time, they developed nocturnal traits that have been observed in other birds that evolved nocturnality independently, such as kiwis and oilbirds. This culminated in a selection of features that make owls uniquely suited to fill the nocturnal predator niche, including rod-rich retinas for better night vision, asymmetrical ears and facial disks for enhanced hearing, and soft feathers that enable silent flight.

**Fig. 1.**
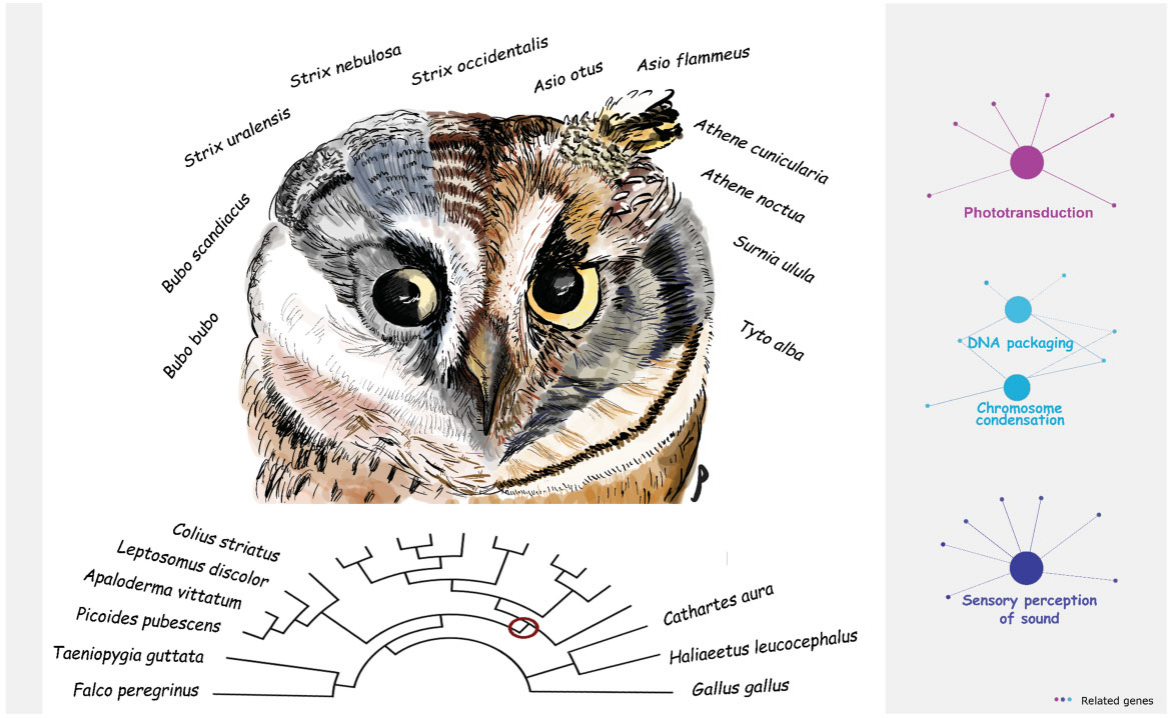
—Genome-wide analysis of 11 owl species reveals that positive selection on phototransduction, acoustic perception, and DNA packaging genes contributed to the nocturnal, predatory lifestyles of owls. Credit: Yifan Pei (drawing) and Pamela Espíndola-Hernández.

In order to identify the evolutionary forces contributing to this confluence of traits, Espíndola-Hernández et al. (2020) compared the genomes of 20 bird species, including 11 owls (five of which were newly sequenced for the study) and analyzed the nucleotide substitution rates of individual genes to identify those that experienced positive selection during the evolution of the owl clade (fig. 1).

As predicted, a primary finding of the study was that genes involved in sensory perception showed a genome-wide signal of positive selection. This category included genes involved in acoustic and light perception, photosensitivity, phototransduction, dim-light vision, and the development of the retina and inner ear. Another set of genes was associated with the plasma membrane, which may reflect the fact that sensory perception depends on signaling cascades that begin on the plasma membranes of photoreceptors. Genes involved in circadian rhythms, which regulate the body’s internal clock, also showed evidence for accelerated rates of evolution, as did some genes related to feather production.

Although these findings were expected, the analysis revealed another category of genes that was wholly unexpected: 32 genes related to DNA packaging and chromosome condensation exhibited an accelerated substitution rate at the origin of the owl lineage. As a putative explanation for this surprising result, the authors point out that the rod photoreceptor cells in the retinas of nocturnal mice and primates exhibit an unusual, radially inverted pattern of heterochromatin and euchromatin. This acts as a sort of collecting lens and increases light detection in the deep layers of the retina. The study’s findings may therefore indicate that owls independently evolved a similar DNA packaging mechanism in the retina that enhances light channeling in photoreceptors, a feature that has not been observed in any bird species to date. If confirmed, this would suggest that selection has acted on the epigenetic mechanisms involved in mediating chromatin structure.

An important caveat to this work is that the findings rely on the accuracy of the functional gene annotations within the owl lineage. Espíndola-Hernández notes that “this is an important challenge for any omics-based research in nonmodel organisms. Although overrepresentation analysis is a powerful tool for functional interpretations of the outcome of different sorts of omics-based tests, its value depends on the quality of the underlying functional databases.” Thus, Espíndola-Hernández hopes to verify the existence of these light-channeling chromatin structures in the owl eye by studying owl photoreceptor cells. Direct investigations like these are critical, Espíndola-Hernández points out, to validate the findings of computational research. She also notes that the study “uses the ratio between the synonymous and nonsynonymous substitution rates, an approach that is based on the assumption that synonymous substitutions are selectively neutral. However, there is evidence that this is not always the case, that is, that these substitutions are not necessarily silent and can be under purifying selection. This crucial point needs to be further explored in the avian lineage.” 
